# Editorial

**Published:** 1868-04

**Authors:** 


### EDITORIAL.

We have just received from the Secretary of the Iowa State Medical Society, Dr. A. G. Field, of Des Moines, a notice announcing the annual meeting at the city of Des Moines, on the fifth day of February, at 10, A. M. It will be remembered that this meeting was appointed for the 5th of February in order that the Society might be in session during the meeting of the Legislature. We hope there may be a full attendance, as matters of interest will be before the Society for their consideration. There are subjects connected with our profession requiring legislative enactments. Let us come together, and by united and harmonious effort much good may be accomplished. The Secretary informs us that he has arranged with the several railroad companies and stage lines to return delegates at one-fifth the regular fare. The profession of Des Moines tenders us a hearty welcome. Let us have a full and profitable meeting and a pleasant re-union.

The Medical Department Iowa University.—We are pleased in being able to announce to the profession of our State the very prosperous condition of this department of the University. The session of 1867–'68, now in progress, has been marked by unusual interest. The *Clinics*, both Medical and Surgical, are more numerous, and of greater interest, than in any former session, and the class giving evidence of rapid advancement. This, to those of us who have struggled since 1850 in the interests of the Medical Department as teachers, is certainly encouraging; and we feel gratified in being able to say that no Western School occupies a prouder position among the regular Schools of Medicine in this country than our own.

EXCHANGES.—The fact that our first issue was an experiment, and contained but little of medical interest, having been used more particularly as a College Circular, we did not present it for exchange to the Medical Journals of the country. Its publication now being a fixed fact, we most cordially ask our own and foreign journals to exchange with us. It is the only Medical Journal of the State, and shall always be found ready to battle for the interests of science, the profession, and its institutions.

---

THE JOURNAL.—Let me, in conclusion, ask each member of the profession to become a subscriber to THE JOURNAL. The amount is small—pay up, and you will have the satisfaction of knowing that you are aiding in a home enterprise. This done, give us an article for publication. Let it be short, and to the point. It will do you good—it will interest others. All we ask is an amount sufficient to defray the expense of publication. This done, we will give you a Bi-Monthly of 64 instead of 32 pages.

---

PROF. G. W. HALL, M. D.—Our new Professor on Physiology and Pathology is winning laurels for himself as a teacher and lecturer in his department.

---

EYE AND EAR.—It will be seen by reference to notice on cover, that a private course of instruction, consisting of twenty lectures, will be given on diseases of these organs, commencing March 3d, 1868, to which the attention of the profession is respectfully invited.

---

HAMLIN'S PATENT PRESCRIPTION CASE.—We have received from Peters & Co., Ottumwa, Iowa, manufacturers, agents, &c., of Hamlin's Prescription Case, one of those very convenient and almost indispensable pocket cases. It is neatly gotten up, and certainly answers the purpose for which it is intended better than anything of the kind before presented to the profession.

JOURNALS RECEIVED.—We welcome first in the list of exchanges with the IOWA MEDICAL JOURNAL, the *American Journal of the Medical Sciences*, for January, 1868, edited by Isaac Hays, M. D., Philadelphia. We are also in receipt of the *St. Louis Medical and Surgical Journal*; the *Medical Record*, New York; the *Boston Journal of Chemistry*, and the *Journal of Materia Medica.*

---

THE LIST OF HONOR.—The want of room prevents us from inserting the names of those who have responded so promptly by sending their subscriptions for THE JOURNAL. We will give their names, with others who will follow their example, in the next issue.

